# World AIDS Day — December 1, 2017

**DOI:** 10.15585/mmwr.mm6647a1

**Published:** 2017-12-01

World AIDS Day, observed each year on December 1, draws attention to the status of the human immunodeficiency virus/acquired immunodeficiency syndrome (HIV/AIDS) epidemic worldwide. The first cases of AIDS were reported in the June 5, 1981, issue of *MMWR* (*1*). Today, approximately 36.7 million persons worldwide are living with HIV infection, including approximately 1.8 million persons who were newly infected during 2016 (*2*). Although the number of annual AIDS-related deaths has declined 48% since 2005, an estimated 1 million persons worldwide died from AIDS in 2016 (*2*).

In the United States, approximately 39,800 persons received a diagnosis of HIV infection in 2016 (*3*). In 2014, an estimated 1.1 million persons in the United States were living with HIV infection, and 85% were aware of their infection (*4*).

Global efforts, including the U.S. President’s Emergency Plan for AIDS Relief, for which CDC is an important implementing agency, resulted in 19.5 million persons worldwide receiving antiretroviral therapy for HIV infection in 2016, an increase from 17.1 million in 2015 (*5*).
